# Disability and Long-Term Care in Singapore

**DOI:** 10.1093/geroni/igaa057.2476

**Published:** 2020-12-16

**Authors:** Cynthia Chen Huijun, Ngee Choon Chia

**Affiliations:** National University of Singapore, Singapore, Singapore

## Abstract

Public systems for long term care (LTC) redistribute resources between generations. Population aging is one of the most significant transformations in the 21st century, where the number of older persons aged 60 years and above is expected to double by 2050, rising to 2.1 billion. We used the Future Elderly Model (FEM) to project the impact of population aging in Singapore up to the year 2050. The FEM is a dynamic economic and demographic microsimulation model. By 2050, the total number of older adults with potential limitation in activities of daily living (ADL) was projected to increase to 275 thousand (18.9%). With the increasing prevalence of disability and chronic diseases, older adults might not have sufficient savings to meet future needs sustainably, despite the expansion of disability insurance from ElderShield to CareShield Life. Lessons and best practices for LTC could be transferred from our experiences to other aging cities globally. Part of a symposium sponsored by International Comparisons of Healthy Aging Interest Group.

